# Dysregulation of tRNA methylation in cancer: Mechanisms and targeting therapeutic strategies

**DOI:** 10.1038/s41420-024-02097-x

**Published:** 2024-07-17

**Authors:** Wenbin Yuan, Rui Zhang, Hao Lyu, Shuai Xiao, Dong Guo, Qi Zhang, Declan William Ali, Marek Michalak, Xing-Zhen Chen, Cefan Zhou, Jingfeng Tang

**Affiliations:** 1 National “111” Center for Cellular Regulation and Molecular Pharmaceutics, Key Laboratory of Fermentation Engineering (Ministry of Education), Cooperative Innovation Center of Industrial Fermentation (Ministry of Education & Hubei Province), Hubei Key Laboratory of Industrial Microbiology, School of Life and Health Sciences, Hubei University of Technology, Wuhan, China; 2 Department of Biological Sciences, University of Alberta, Edmonton, AB Canada; 3 Department of Biochemistry, University of Alberta, Edmonton, AB Canada; 4 Membrane Protein Disease Research Group, Department of Physiology, Faculty of Medicine and Dentistry, University of Alberta, Edmonton, AB Canada

**Keywords:** Oncogenes, Drug development

## Abstract

tRNA is the RNA type that undergoes the most modifications among known RNA, and in recent years, tRNA methylation has emerged as a crucial process in regulating gene translation. Dysregulation of tRNA abundance occurs in cancer cells, along with increased expression and activity of tRNA methyltransferases to raise the level of tRNA modification and stability. This leads to hijacking of translation and synthesis of multiple proteins associated with tumor proliferation, metastasis, invasion, autophagy, chemotherapy resistance, and metabolic reprogramming. In this review, we provide an overview of current research on tRNA methylation in cancer to clarify its involvement in human malignancies and establish a theoretical framework for future therapeutic interventions targeting tRNA methylation processes.

## Facts

- tRNA methylation is crucial for cancer cell growth.
- tRNA methylation enhances cancer cell growth and chemoresistance by promoting the translation of oncogenes, autophagy, and change of tumor microenvironment.
- Inhibition of the tRNA methylation process is a potential strategy for cancer therapy.

## Open questions

- Does tRNA methylation always promote cancer cell growth?
- What is the molecular mechanism underlying the upregulation of tRNA methylation in cancer cells?
- How can drug design be optimized to selectively target the tRNA methylation process in cancer cells?

## Introduction

Transfer RNAs (tRNAs) are small, noncoding RNA molecules that facilitate the decoding of mRNA codons and transport amino acids to ribosomes during protein synthesis. Previously, tRNA was solely believed to be involved in cellular amino acid transportation. With the rapid advancements in technologies such as sequencing and mass spectrometry, the enigma surrounding tRNA modification regulatory mechanisms in tumor cells is gradually being unraveled, extending beyond its involvement in amino acid transportation. The multifaceted functions of tRNA have been implicated in various diseases, encompassing tumor metabolism, immune response modulation, and the intricate tumor microenvironment [1, 2]. The tRNA modifications are the most abundantly among modified RNAs, with over 100 types of modifications have detected in tRNA [1]. tRNA modification facilitates the improvement of tRNA stability, the ability of tRNA to transport specific amino acids as well as the efficiency of protein synthesis [3]. The modification of tRNA drives protein translation, leading to an increased level of translation for a specific class of proteins, thereby contributing to the proliferation of cancer cells [4]. In addition, tRNA modification and stability can regulate the metabolic state of cancer cells. The main types of post-transcriptional modifications of tRNA include methylation, deamination and acetylation, which methylation is the most common tRNA modification type and also a hot spot for research in recent years [5]. This implies that modulating the methylation process of tRNA holds promising implications for anti-tumor therapy.

Cancer cells typically exhibit heightened proliferation and metabolic rates compared to normal cells, necessitating the synthesis of a substantial number of oncogenic proteins to sustain cancer cells growth [6]. Consequently, the homeostasis of tRNA assumes particular significance in cancer cells, wherein methylation modifications enhance its efficacy. tRNA methylation plays a pivotal role in facilitating tumor growth; however, limited research has been conducted on the therapeutic targeting of this highly efficient and specific mechanism. In this review, considering the crucial role of tRNA methylation in cancer cell survival, we comprehensively summarize the latest advancements in tRNA methylation research and emphasize the underlying molecular mechanisms through which tRNA methylation facilitates cancer cell survival while proposing novel insights. Furthermore, in light of the dominant obstacles encountered in cancer therapy like resistance to chemotherapy drugs, inaccurate targeting, and limitations of surgery, this research advocates for an innovative treatment strategy focusing on inhibiting the tRNA methylation pathway in cancerous cells. Currently, there are few studies or reviews reported on therapeutic approaches targeting the tRNA methylation process. This innovative strategy not only offers valuable insights but also holds significant potential for advancing drug discovery, development, and clinical treatment to address the persisting issue of cancer therapy. Moreover, the assessment of tRNA methylation status in cancer cells can serve as a pivotal diagnostic and prognostic biomarker for cancer, facilitating personalized treatment strategies for clinical practitioners.

## tRNA biological function

### Mechanism of cytoplasm tRNA biosynthesis and transport

**Fig. 1:**
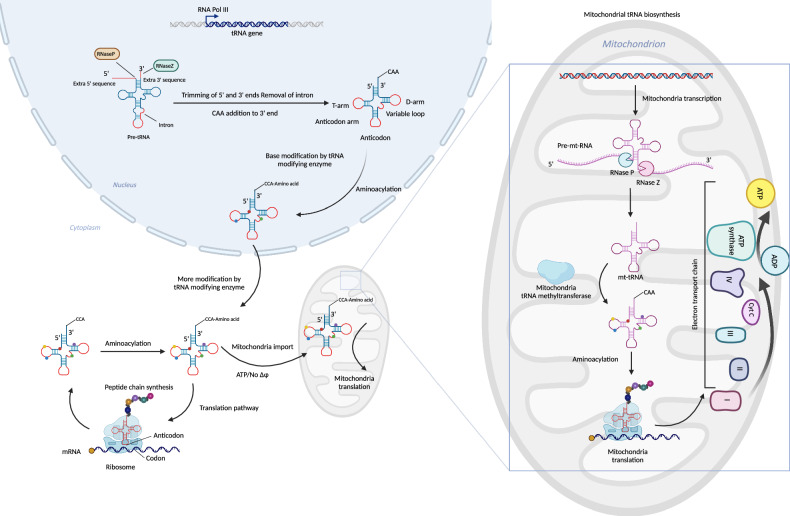
The process of nucleus tRNA and mitochondrial tRNA biosynthesis. The mechanisms of nucleus tRNA biosynthesis involves DNA polymerase III recognizing and binding to the promoter region of tRNA genes on the genomic DNA to initiate the transcription of precursor tRNA (pre-tRNA). This transcription process produces a primary pre-tRNA product containing additional sequences at both the 5’ and 3’ ends. After undergoing a series of cleavage and modification steps, the tRNA is released from the nucleus into the cytoplasm. In the cytoplasm, tRNA is further modified and aminoacylated to form mature tRNA, which participates in ribosome-mediated protein translation. Some of the mature tRNAs are imported into mitochondria to be involved in mitochondrial protein synthesis; Mitochondrial tRNA biosynthesis involves transcription of mitochondrial tRNA genes from mitochondrial DNA, followed by cleavage mediated by RNase P and RNase Z within mitochondria to generate mitochondrial tRNA. Subsequently, these tRNAs undergo modifications and aminoacylation catalyzed by mitochondrial tRNA-modifying enzymes, ultimately participating in intra-mitochondrial protein translation. Created with BioRender.com.

Typically, the function of tRNA is to carry amino acids and recognize codons in the CDS region of mRNA to synthesize proteins in the ribosome according to the sequence of mRNA. tRNA in the cytosol is composed of 73–93 nucleotides, while mitochondrial tRNA(mt-tRNA) has only 57 nucleotides [7]. In human cells, 610 genes encoding tRNAs and mitochondrial tRNAs have been identified [8]. The tRNA is transcribed from the genome by RNA polymerase III (Pol III) and is cleaved by RNase P and RNase Z at the 5’ and 3’ trains to form the primary pre-tRNA [9]. The pre-tRNA is catalyzed by tRNA nucleotide transferase, which adds the CCA sequence at the end of 3’, and then exits the nucleus after the initial tRNA modification by tRNA-modifying enzymes in the nucleus. With the help of NPC proteins, tRNA exits the nucleus into the cytoplasm and is further processed by tRNA-modifying enzymes in the cytoplasm to form a mature and stable tRNA. The mature tRNA secondary structure is a cloverleaf shape with T-loop, D-loop, V-loop, and amino acid arms, while the tRNA tertiary structure (3D) is an inverted triangle [10] (as shown in Fig. 1).

### Mechanism of mitochondrial tRNA biosynthesis and import

The tRNA exiting the nucleus serves two distinct functions: one is to carry free amino acids in the cytosol for ribosome-mediated protein synthesis, utilizing mRNA as a template; and the other is to enter the mitochondria and actively participate in mitochondrial life processes.

Mitochondria, as a semi-independent organelle, have their own genetic system and independent protein synthesis system which can also transcribe tRNA independently. Mitochondrial DNA encodes 22 tRNA genes, and mt-mRNA and mt-rRNA genes are flanked by tRNA cloverleaf structures that act as recognition elements for nuclear cleavage, which can release a single mtRNA molecule cleaved by RNase Z and RNase P. At the same time, mtRNA is modified by the modification enzymes in the mitochondria and participates in the synthesis of mitochondrial proteins to maintain mitochondrial metabolism [11].

However, even though the mitochondria can transcript tRNA independently, the limited number of tRNA genes in mitochondrial DNA hinders the adequate synthesis of mitochondrial proteins. Therefore, the import of nucleus-encoded tRNAs from the cytoplasm into mitochondria is crucial for their participation in mitochondrial translation. The mechanism underlying the translocation of cytoplasmic tRNA into mitochondria remains elusive. In human cells, it is currently postulated that tRNA can translocate into mitochondria via ATP-dependent import mechanism without perturbing the membrane potential [12, 13].

The mitochondria autonomously synthesize 13 proteins and interact with an additional 84 nuclear-encoded proteins to form respiratory chain complexes that augment ATP production. tRNA plays a pivotal role in mitochondrial translation, thus making the mitochondrial protein synthesis pathway involving mitochondrial tRNA crucial for maintaining cancer cell respiration and energy generation [14] (as shown in Fig. 1).

### Mechanisms of tRNA-derived fragments biosynthesis and function

**Fig. 2:**
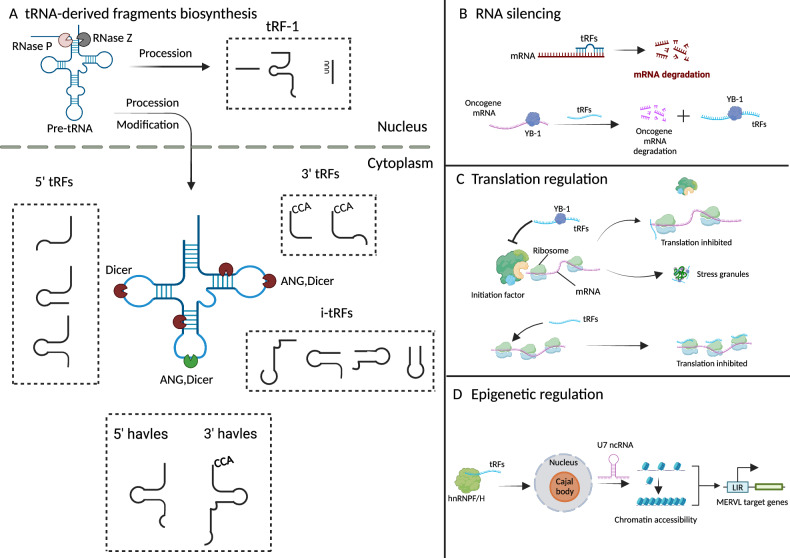
tRNA-derived fragments biosynthesis, categorization and function. **A** The tRF-1 series is generated through the cleavage of pre-tRNA by RNase Z (or ELAC2) during tRNA processing in nucleus. According to the incision site of mature tRNAs, tRFs can be categorized into 5’-halves, 3’-halves, 5’-tRFs, 3’-tRFs, and i-tRFs. **B** RNA silencing : The specific tRFs mediate RNA silencing by interacting with the 3′-UTR regions of mRNA or combining with YB1 to degrade oncogene mRNA [25, 26, 207, 208]. **C** Translation regulation: tRFs can inhibit global translation by promoting stress granule assembly through binding with YB-1, or tRFs can directly bind to ribosomes, affecting ribosome assembly or function, thereby regulating translation [209, 210]. **D** Epigenetic regulation: tRFs directly bind to heterogeneous nuclear ribonucleoproteins F and H (hnRNP F/H), promoting cajal body biogenesis by enhancing histone expression levels through U7 snRNA. Elevated histone levels inhibit the expression of MERVL-target genes by altering the chromatin state from euchromatin to heterochromatin [211, 212]. Created with BioRender.com.

The origin of tRNA-derived fragments (tRFs) can be traced back to tRNA. When tRNA is not sufficiently modified, tRNA stability could be disrupted and cleaved to tRFs or degraded by Dicer, endonuclease Z (RNase Z/ELAC2) and other RNA endonucleases (RNases) [15–17]. tRFs/tiRNAs are categorized into five types based on their cleavage sites on mature tRNA by different nucleases, including 5’-half, 3’-half, 5’-tRF, 3’-tRF, and i-tRF [18–20]. Among them, 5’-half and 3’-half are usually produced under various stress conditions in cells, generated by the activation of angiogenin (ANG) enzyme activity, which cleaves tRNA at the anticodon site [21, 22]. The generation mechanism of 3’-tRF primarily involves nucleases such as Dicer and ELAC2 (RNase Z) cleaving at the 3’ terminus of tRNA, while the generation mechanism of 5’-tRF mainly entails nucleases such as Angiogenin cleaving at the 5’ terminus of tRNA [23]. The main factor contributing to the production of i-tRFs (internal tRNA-derived fragments) is the activity of specific nucleases, which cleave the tRNA molecule at specific locations such as the anticodon loop or D-loop [24, 25]. Additionally, tRF-1 represents a noncanonical class of tRFs generated from pre-tRNAs. tRF-1 is predominantly derived from the 3’ trailer fragment of precursor tRNA, processed either by RNaseZ or its cytoplasmic homolog, ELAC2 [26, 27] (as shown in Fig. 2A). tRFs was initially thought to be the product of random degradation of tRNA. Mounting evidence has substantiated the functional role of tRF and its involvement in diverse human diseases, including human cancers. tiRNAs/tRFs can be divided into three major categories based on their biological functions in the cell, including epigenetic regulation, RNA silencing, and translation regulation [28] (as shown in Fig. 2B–D). Despite limited understanding of tRF biogenesis and functions, emerging findings suggest that the methylation status of tRNA not only impacts its stability and function but also governs the process of tRF biogenesis [29]. However, with the continuous discovery of the function of tRNA in cancer in recent years, tRF has expanded the function of tRNA and played an important role in the process of various cancers, which has become a hot spot in the field of tRNA research [28].

### The methylation modification of tRNA in cancer cells

The expression of tRNA is dysregulated in cancer cells, which is intricately associated with tRNA modification. tRNA represents one of the most abundantly modified RNAs, and its modification plays a pivotal role in translation accuracy, translation efficiency, and changing in intracellular tRNA abundance [30]. The evidence of the tRNA regulation mechanism of tumor growth has just been found that regulate tRNA metabolism and enhance the protein synthesis rate in response to vigorous metabolic demand of tumor cells [31, 32], and promote the proliferation of cancer cells, increase protein synthesis efficiency. However, increasing mRNA translation speed can reduce protein translation accuracy [33]. High levels of proteasome activity and activation of autophagy pathways are essential to promote the survival of tumor cells under environmental and immune pressures [34]. Therefore, tRNA modification has been recognized as an important regulatory factor in cancer cells.

The current studies have shown that although tRNAs are mainly involved in regulating the translation process of mRNAs by ribosomes, the function of tRNAs in the cytoplasm differs from that of tRNAs in mitochondria in terms of the mechanism of cancer cell promotion. For example, in the cytoplasm, abnormal tRNA modifications mainly mediate the translation of oncogenes, for example, Methyltransferase 1/WD Repeat Domain 4 (METTL1/WDR4)-mediated m^7^G tRNA modifications can enhance the translation of CyclinD1, a recognized proto-oncogene, in head and neck squamous cell carcinoma, and CyclinD1 overexpression can lead to uncontrolled cell proliferation [35]. However, aberrant tRNA modification in mitochondria enhances mitochondrial function in tumor cells by facilitating translation of mitochondrial ribosomes and promoting the adaptive capacity of tumor cells to survive in a malignant microenvironment. Insufficient modification of mitochondrial tRNA leads to the formation of non-functional secondary structures, thereby impairing mitochondrial function [36–38].

**Fig. 3:**
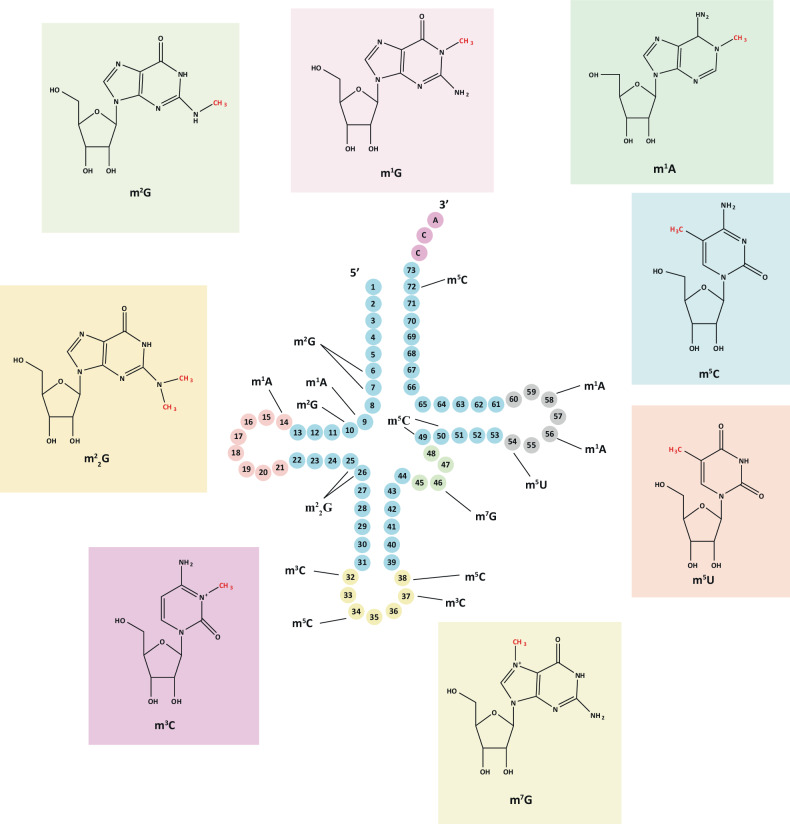
tRNA methylation modification sites and species. The annotation for tRNA methylation modification is as follows: m^1^G 1-methylquanosine, m^2,2^G N2, N2-dimethylguanosine, m^2^G N2-methylquanosine, m^1^A N1-methyladenosine, m^5^C 5-methylcytidine, m^5^U 5-methyluridine, m^7^G 7-methylguanosine, m^3^C 3-methylcytidine.

More than 80 tRNA modifications have been identified, with an average of 13 modifications per tRNA molecule [39]. RNA methylation modification is a common post-transcriptional modification, and tRNA also has a very wide type of methylation modifications, including m^1^A, m^5^C, m^7^G, etc. The methylation modification of tRNA is inseparable from the function of tRNA methyltransferase. tRNA methyltransferase can methylate specific sites of tRNA to promote the stability of tRNA and change the ability of amino acid deliver and ribosome occupancy of different tRNAs [40]. The expression levels of tRNA methyltransferases are significantly upregulated in various types of tumor cells and patients, indicating the crucial involvement of tRNA methylation in tumor progression [38] (As shown in Table 1). Meanwhile, methylation modification is the most important and studied type of tRNA modification in tumor biology at present [41] (as shown in Fig. 3).

**Table 1:** tRNA methyltransferase in human cancers.

tRNA modification	Regulators	Role	tRNA modification site	Distribution	Cancer type	Reference
m^1^A	TRMT6/TRMT61A	Oncogene	56	Cytoplasm/nucleus	Hepatocellular Carcinoma	[179]
					Bladder cancer	[180]
	TRMT61B	Oncogene	58	Cytoplasm/mitochondrial	Hepatocellular Carcinoma	[179]
	TRMT10C	Oncogene	9	Cytoplasm/mitochondrial	Hepatocellular Carcinoma	[179, 181]
m^3^C	METTL2A	Oncogene	32,37	Cytoplasm	Pancreatc cancer	[66]
					Breast Cancer	[68]
	METTL2B	Oncogene	32	Cytoplasm	Pancreatc cancer	[66]
	METTL6	Oncogene	32	Nucleus/cytoplasm	Pancreatc cancer	[66, 182]
					Hepatocellular Carcinoma	[70]
	METTL8	Oncogene	32	Mitochondrial	Pancreatc cancer	[64, 183]
m^7^G	METTL1	Oncogene	46	Nucleus	Glioblastoma	[184]
					Melanoma	[185]
					Lung cancer	[83]
					Hepatocellular Carcinoma	[155]
					AML	[4, 186]
					Head and Neck squamous cell carcinoma	[35]
					Nasopharyngeal Carcinama	[82]
m^5^C	NSUN2	Oncogene	34,48,49,50	Mitochondrial/nucleus	Anaplastic thyroid cancer	[88, 92, 187, 188]
	NSUN3	Oncogene	34	Mitochondrial	Esophageal cancer	[96, 189]
					Head and Neck squamous cell carcinoma	
	NSUN6	Oncogene	72	Cytoplasm	Pancreatic cancer	[97, 190, 191]
	DNMT2	Oncogene	38	Cytoplasm	Prostate Cancer	[109, 192, 193]
					Breast Cancer	[194]
m^5^U	TRMT2A	Tumor suppressor	54	Nucleus	Breast Cancer	[98, 195, 196]
m^2^G	THUMPD3	Uncertain	6,7	Cytoplasm	Colon cancer	[103, 105]
	TRMT11	Uncertain	10	Cytoplasm	Colon cancer	[105]

#### N1-methyladenosine (m^1^A)

m^1^A is a universal RNA modification that modifies a methyl group on the first nitrogen atom of adenosine in RNA [42]. m^1^A in tRNA adenosine occurs at the Watson-Crick interface and may affect RNA basal pairing [43]. Currently, m^1^A is found in a variety of RNAs, including tRNA, mRNA, rRNA, and some LncRNAs, and m^1^A is most modified in tRNA, while in mRNA, m^1^A modification is less prevalent, so m^1^A modification of tRNA has received a lot of attention from researchers in recent years. The present study reports that m^1^A modification occurs at positions 9, 14, 22, 57, and 58 in cytoplasmic tRNAs, and at positions 9 and 58 in mitochondrial tRNAs [44]. tRNA regulators of m^1^A modification in mammalian cells are mainly Writer (TRMT6, TRMT61A, TRMT61B TRMT10C) and eraser (ALKBH1, ALKBH3, ALKBH7, FTO), while the readers in tRNAs are not well understood now [42, 45]. In mitochondria, TRMT10C, TRMT61B, and ALKBH7 are mainly responsible for the m^1^A modification and de-modification of mitochondrial tRNA, while TRMT6.TRMT61A, ALKBH1, and ALKBH3 are distributed in the cytoplasm and mainly responsible for the modification and de-modification of tRNA from nucleus [42, 45]. Among the modification sites of m^1^A, m^1^A58 is an important and highly conserved modification and m^1^A modification occurs in all processes of life [46]. m^1^A was catalyzed mainly by TRMT6 and TRMT61A [47], while TRMT61B specifically catalyzed tRNA m^1^A 58 site [48]. m^1^A 58 can be demethylated by ALKBH1 and ALKBH1 can be demethylated in cytosolic and mitochondrial tRNAs [45, 49]. m^1^A58 is essential for the initiation of protein translation, and ALKBH1-mediated deletion of m^1^A58 modification leads to degradation of tRNA^iMet^, which inhibits the translation efficiency of the protein, and resulted in a slow cell growth phenotype with low modification of m^1^A58 [50, 51]. This also suggests that ALKBH1 may be a potential tumor suppressor gene. However, ALKBH1 has been implicated in the promotion of tumorigenesis across various cancer types, potentially attributed to its demethylase activity that extends beyond tRNA and encompasses other RNA or DNA substrates [52–54].

Dysregulation of the level of tRNA m^1^A modification is associated with a variety of diseases including cancer, such as dysregulation of m^1^A tRNA modification in mitochondria and cytoplasm in AD [55], However, recent studies have unveiled the escalating significance of tRNAs harboring aberrant m^1^A modifications in the realm of cancer research. For instance, a recent study demonstrated that hepatocellular carcinoma exhibits abnormally elevated levels of m^1^A, which is associated with a poor prognosis for patients, while TRMT6/TRMT61A is the main enzyme mediating this aberrant m^1^A modification, m^1^A56 modification of tRNA^Ala^-AGC and tRNA^Glu^-CTC promotes the translation of Peroxisome Proliferator Activated Receptor Gamma (PPARδ) and thus enhances the lipid metabolism of hepatocellular carcinoma cells and thus promoting the progression of hepatocellular carcinoma [56]. Cancer cells have more active lipid metabolism than normal cells, which can promote the spread of tumor [57]. Additionally, the expression of ALKBH3, a demethylase responsible for tRNA m^1^A modification, was significantly downregulated in cancer cells compared to normal cells. Notably, hypermethylation of the ALKBH3 promoter CpG island has been observed in Hodgkin’s lymphoma, resulting in impaired intracellular demethylation capacity of tRNA m^1^A. Consequently, this dysregulation affects the translation of migration and cytoskeleton-related genes associated with poorer prognosis [58]. Overall, those evidence suggests that tRNA m^1^A modification levels are abnormally upregulated in a variety of tumor cells, enhancing cancer cell metabolism and promoting cancer cell survival.

#### N3-methylcytidine (m^3^C)

N3-methylcytidine(m^3^C) means that the methyl group is attached to the third carbon of the cytosine base. In DNA, m^3^C occurs primarily on single-stranded DNA, while in RNA, m^3^C is mediated by SAM-dependent methyltransferase [59, 60]. At the same time, m^3^C was also repeatably detected outside the tRNA anticodon ring, predominantly at the 32 site [61, 62]. It has been shown that m^3^C32 helps maintain the anticodon loop in the optimal conformation for tRNA function [63]. m^3^C modification only affects nascent tRNAs, and its effect on periodic tRNAs is not obvious [64]. The m^3^C methyltransferases identified in human cells are METTL2A/METTL2B (methylated mainly at tRNA^Thr^AGU, tRNA^Thr^UGU and tRNA^Arg^CCU), METTL6 (methylated mainly at tRNA^Ser^AGA, tRNA^Ser^CGA, tRNA^Ser^UGA, tRNA^Ser^GCU) and METTL8 (methylated mainly at tRNA^Thr^UGU). Interestingly, almost all of them are methylated at 32 site in cytidine [64, 65] .Upregulation of m^3^C levels was observed in different cancers. For example, the m3 C32 level of mt-tRNA^Ser^ in pancreatic cancer cell line PNAC-1 was significantly increased compared with normal pancreatic cells [64], which is associated with upregulation of m^3^C methyltransferase in cancer.

METTL2A and METTL2B are two homologs of METTL2, which differ in only 6 amino acids. In some eukaryotic cells, METTL2 is the only form, such as mice. In human cells, METTL2A and METTL2B coexist and share the same cell distribution, both located in the cytoplasm. However, the m^3^C catalytic activity of METTL2B was much lower than that of METTL2A [66, 67]. G35 and t6A37 are essential for METTL2A to catalyze tRNA^Thr^ m^3^C modification at position 37 of the tRNA anti-codon ring. Compared with METTL2A, METTL2B has little catalytic modification activity on m^3^C32. Recent studies have shown that METTL2A is a potential oncogene in breast cancer, and high expression of METTL2A leads to elevated levels of m^3^C modification associated with cell proliferation and activation of the pathway [68]. Furthermore, an additional study demonstrated that METTL2B serves as a prominent biomarker in gastric cancer and exhibits a strong correlation with unfavorable prognosis, thereby indicating the potential of METTL2B for assessing cancer progression [69].

In contrast to METTL2A/2B, the interaction with seryl-tRNA synthetase (SerRS, encoded by SARS1) is essential for METTL6, facilitating the biogenesis of m^3^C32 modifications in human tRNA^Ser^. Consequently, METTL6 exhibits distinct substrate selection preferences compared to METTL2A/2B [66]. However, the depletion of METTL6 only halved the m^3^C level in these tRNA^Ser^ homoreceptors, suggesting that the second m^3^C site in the variable loop of these tRNA homoreceptors may be methylated by another unknown enzyme [67]. METTL6 functions as a m^3^C methyltransferase, exerting regulatory control over tumor cell proliferation. Deletion of METTL6 significantly impacts both mRNA expression and translation levels, leading to cellular growth defects and compromised pluripotency. Furthermore, METTL6 knockout mice showed a metabolic disorder phenotype [70]. These findings suggest that METTL6-mediated methylation of m^3^C tRNA may have a novel regulatory mechanism for gene expression, translation, cell homeostasis, and tumor cell growth. Subsequent studies have shown that down-regulating METTL6 slows the progression, migration, invasion, and adhesion of hepatoma cells by inhibiting cell adhesion molecules [71]. Knockdown of METTL6 significantly reduced the sensitivity of lung cancer cells to cisplatin [72]. Although many studies have reported that METTL6 is involved in cancer progression, but the mechanism remains unclear.

METTL8 has been identified as an RNA m^3^C methyltransferase, but it is controversial whether METTL8 is mainly involved in the modification of mRNA or tRNA. The limited effect of METTL8 knockout on tRNA abundance seems to support the hypothesis that METTL8 is mRNA m^3^C methyltransferase rather than tRNA methyltransferase [67]. However, subsequent studies have shown that METTL8 is a mitochondria-specific tRNA m^3^C methyltransferase [73] that balances mitochondrial translation by METTL8-mediated m^3^C modification of mitochondrial tRNA [64]. However, METTL8 has been identified as an oncogene in cancer, and a recent report showed that METTL8 knockdown inhibits breast cancer cell growth and strongly blocks breast cancer cell migration [74]. In addition, the SUMOylation of METTL8 induced tRNA R-loop formation and promoted colorectal cancer tumorigenesis [75]. Even though the role of METTL8 in cancer has been confirmed, whether METTL8 is dominated by mRNA or m^3^C modification of mt-tRNA in cancer cells still needs to be further studied.

The study of tRNA m^3^C demethylases is still not very clear, but there is a small amount of evidence pointing to demethylation of tRNA m^3^C, but the reversion of tRNA m^3^C levels by ALKBH3 deletion is not very significant, while ALKBH3 supports tumor cell growth and is localized in the nucleus, which makes whether ALKBH3 regulates tumor cell growth through tRNA m^3^C demethylation-dependent manner is still one of the scientific questions worth investigating [76–78].

#### N7-Methylguanosine (m^7^G)

N7-Methylguanosine(m^7^G) is positively charged and is produced by the addition of methyl groups at the N7 site of guanosine. This is the most prevalent mRNA cap modification and is also present in internal mRNA, microRNA, tRNA, and rRNA [79]. The tRNA 46 guanosine position (m^7^G46) is the most common m^7^G methylation site [80]. The m^7^G46 is located in the variable loop region in tRNA [81], and the m^7^G46 modification in the variable loop region forms a tertiary base pair with C13-G22, which stabilizes the tRNA structure [81]. Moreover, the modification of m^7^G on tRNA is predominantly facilitated by the METTL1/WDR4 complex [79].

The role of m^7^G in cancer has received increasing attention in recent years. m^7^G methylation modification is also one of the most studied tRNA methylation modifications and has been reported to be highly expressed in a variety of cancers. The identification of METTL4/WDR4 as an oncogene involved in m^7^G tRNA methylation has been reported [4]. METTL1 deletion leads to decreased methylation and expression of m^7^G modified tRNA, global translation obstruction, and cell cycle defects. METTL1 knockdown inhibits the growth of a variety of tumors, including glioblastoma, melanoma, and AML. Overexpression of METTL1/WDR4 leads to malignant transformation and tumorigenesis, promote cancer progression, and may be highly associated with m^7^G methylation modification of tRNA^Arg^TCT-4-1 [4]. The incidence of nasopharyngeal carcinoma is high, and patients with advanced stages have a poor prognosis, indicating its classification as a malignancy with unfavorable outcomes. High levels of METTL1-dependent m^7^G-tRNA codons selectively increase the corresponding mRNA levels, activate the Wnt/β-catenin pathway and promote the EMT process, increase CyclinD1 protein levels in nasopharyngeal carcinoma cells, and promote the proliferation and migration of cancer cells [82]. In a similar way to nasopharyngeal carcinoma, in lung cancer, the METTL1/WDR4 complex selectively promotes cell cycle related mRNA (including Cyclin D3 and Cyclin E1) translation processes via m^7^G tRNA codon dependence [83]. In hepatocellular carcinoma, METTL1/WDR4-mediated m^7^G tRNA modification significantly promotes the translation of cyclin A2, EGFR, and VEGFA, promoting the spread and metastasis of hepatocellular carcinoma cells [84]. In bladder cancer, METTL1-m^7^G-mediated tRNA methylation promotes EGFR/EFEMP1 translation and thus promotes angiogenesis and cancer cell metastasis [85].

In summary, METTL1/WDR4 is the main enzyme complex mediating the methylation of tRNA m^7^G. METTL1/WDR4 has a strong carcinogenic effect in different cancer types, which supports that METTL1/WDR4 is an oncogene. Meanwhile, in cancer cells, METTL1/WDR4 mainly promotes the translation of genes related to cell proliferation, drug resistance, and angiogenesis by mediating m^7^G methylation of tRNA, so that cancer cells can better survive in the environment. These studies suggest that METTL1/WDR4 may be a potential pan-cancer therapeutic target.

#### 5-Methylcytosine (m^5^C)

Similar to the tRNA methylation modification mentioned above, m^5^C modification widely exists in all kinds of RNA. SAM is usually the active methyl group of the donor, which is added to the C-5 position of the cytosine base in the RNA to form the m^5^C modification of RNA [86]. Methylation of tRNA occurs most frequently on cytosines in the junction region between the variable loop and the T-stem, and m^5^C residues are present on eukaryotic tRNA cytosines C34, C38, C48, C49, and C72 [87]. They are involved in the composition of tRNA secondary structure, which is related to codon recognition and stability of tRNA [88]. m^5^C modification of tRNA by m^5^C has been extensively studied in recent years. The m^5^C modification of tRNA has been shown to maintain intracellular metabolic balance, optimize codon-anticodon pairing, and regulate protein translation efficiency and accuracy [89]. Simultaneously, the m^5^C modification of tRNA holds great promise as a potential biomarker for cancer diagnosis [90].

The main m^5^C modification enzymes in tRNA are NSUN family proteins (NSUN2, NSUN3, NSUN6) and DNMT2-regulated m^5^C modification of tRNA. For example, NSUN2-mediated m^5^C tRNA modification can promote drug resistance, differentiation, and self-renewal of cancer cells [91, 92]. At the same time, NSUN2 introduces 5-methylcytosine to mammalian mitochondrial tRNA [88]. It has been reported that NSUN2 is a target of MYC oncogene and drives cell proliferation and growth [93]. NSUN2 is highly expressed in various tumor tissues, including gastric cancer, pancreatic cancer, breast cancer, etc [94].

NSUN3 is involved in the biogenesis of 5-formylcytidine(f^5^C) in human cells. In human cells mitochondria, the AUA codon encodes methionine via the mitochondrial methionine transfer RNA (mt-tRNA^Met^), which contains f^5^C at the first position of the anticodon (position 34). f^5^C34 is required for decoding AUA during mitochondrial protein synthesis codon is required for mitochondrial protein synthesis. To date, the biogenesis mechanism and physiological role of f^5^C34 remain elusive. The biogenesis of f^5^C34 is currently thought to be initiated by s -adenosylmethionine-dependent m^5^C methylation, and NSUN3 is essential in mediating m^5^C modification in mitochondrial tRNA^Met^. mitochondrial protein content is significantly reduced in NSUN3 knockout cell lines, and therefore NSUN3 is considered a potential and important methyltransferase in mitochondria [95]. NSUN3-mediated mt-tRNA m^5^C modification is closely associated with cancer cell metastasis, and a recent study reported that when cancer cells plan to invade and spread, their mitochondrial tRNAs are modified with m^5^C, thus promoting cancer cell metastasis. Cancer cell metastasis is actually heavily dependent on mitochondria for energy, Mitochondria possess independent genetic material that can be used to produce proteins required for the respiratory chain in energy production. When the level of mitochondrial tRNA m^5^C modification in cancer cells is at a low level, glycolysis will be the main energy production pathway of cancer cells, and this level of energy supply is far from sufficient to support cancer cells to metastasize, so high m^5^C modification level is necessary to support cancer cells to metastasize. In contrast, NSUN3-mediated mitochondrial tRNA m^5^C modification is a major factor in cancer cell metastasis. When the expression of NSUN3 was turned off, m^5^C modification of mitochondrial tRNA was also reduced, which was accompanied by a significant decrease in cancer cell invasiveness [96].

The important role of tRNA m^5^C modifications and modifying enzymes in tumor survival is unquestionable. Multiple RNA molecules can be modified by numerous m^5^C methyltransferases, and potential crosstalk between distinct RNA m^5^C methyltransferases may influence the impact of m^5^C on cancer cells. Thus, making it exactly which RNA modifications are dominant for cancer cell survival still lacks strong evidence to support, for example NSUN6 as m^5^C methyltransferase of mRNA and tRNA is significantly upregulated in pancreatic cancer cells and promotes pancreatic cancer cell progression [97], but still no detailed elucidation of which RNAs are most important for NSUN6-mediated m^5^C modifications. However, even so, m^5^C modifications as well as modifying enzymes are very promising targets for tumor therapy as well as tumor markers.

#### Other tRNA methylation modifications

Apart from the extensively studied methylation modification species mentioned above, these modifications have received relatively less attention in the context of cancer research. However, it is undeniable that these tRNA methylation modifications and methyltransferases hold significant implications for the progression of cancer. For example, TRMT2A-mediated 5-methyluridine (m^5^U) tRNA methylation can affect cell proliferation, TRMT2A localizes in the nucleus, possibly modifying tRNA in the nucleus, and TRMT2A inhibits cell proliferation blocking it in the G2/M phase, and knockout TRMT2A in MEF cells can promote MEF cell proliferation, suggesting that TRMT2A-mediated m^5^U tRNA modification may inhibit tumor cell growth [98].

**Fig. 4:**
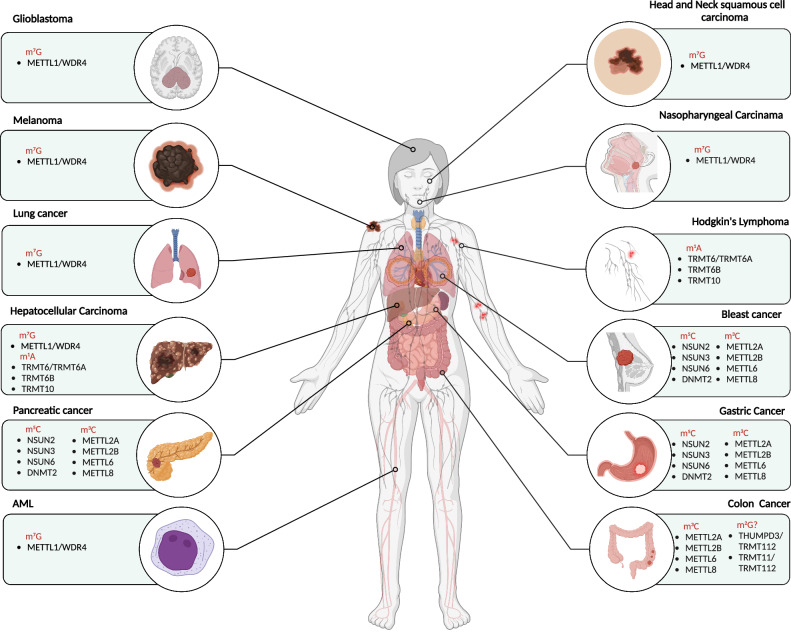
The role of tRNA methylation in human cancer. tRNA m^1^A modification is implicated in the progression of hepatocellular carcinoma and Hodgkin’s lymphoma; The role of tRNA m^2^G modification in colorectal cancer remains uncertain; tRNA m^3^C modification is implicated in the progression of pancreatic cancer, breast cancer, gastric cancer and colorectal cancer; tRNA m^5^C modification is implicated in the progression of pancreatic cancer, breast cancer and gastric cancer; tRNA m^7^G modification is implicated in the progression of glioma, melanoma, lung cancer, hepatocellular carcinoma, Acute myeloid leukemia (AML), head and neck squamous cell carcinoma, and nasopharyngeal carcinoma. Created with BioRender.com.

N2-methylguanine(m^2^G) was formed by aminomethylation of guanine at C2 position [99]. The m^2^G modification of tRNA molecules is widespread and conserved in eukaryotes, archaea and some bacteria [100]. m^2^G modification of tRNA usually occurs at positions 6 and 10 [101]. The m^2^G modification is predominantly mediated by TRMT11 and THUMPD2/THUMPD3, both of which necessitate the assistance of a co-factor, TRMT112, to facilitate their catalytic activity [102, 103]. In yeast cells, loss of Trm11 (TRMT11 homolog) does not affect yeast growth [104]. However, knockout of THUMPD3 in HEK-293T cells resulted in global protein translation impaired and inhibition of cell growth [103]. A study just published showed that knockout THUMPD3 did not affect cell proliferation in human colon cancer cells HCT116, while knockout TRMT11 increased cell proliferation, and interestingly, the proliferation of HCT116 cells was significantly inhibited when both TRMT11 and THUMPD3 were knocked out [105]. This contradictory result may be due to differences in cell lines and may be related to the interaction of TRMT112 with other methyltransferases. However, TRMT112 also exhibits different effects on cell proliferation. In pancreatic cancer, TRMT112 and WBSCR22 synergistically inhibit pancreatic cancer progression by repressing ISG15 transcription, which seems to support the possibility that TRMT112 is a tumor suppressor [106]. However, another report showed that TRMT112 was significantly upregulated in multiple cancers and was accompanied by a poorer prognosis [107]. Therefore, further validation is required to ascertain the involvement of TRMT112 and TRMT11/THUMPD2/THUMPD3 in the proliferation of diverse cancer cells. The functional significance of TRMT11/THUMPD2/THUMPD3-mediated tRNA m^2^G modifications in cancer cells remains largely unexplored compared to extensively investigated modifications such as m^7^G and m^5^C (as shown in Fig. 4).

#### The role of tRNA-derived fragments production regulated by tRNA methylation in cancer cell

tRF plays a dual role in tumorigenesis, with some tRF promoting tumor cell growth while others inhibit it; moreover, different types of tRF exhibit distinct regulatory patterns in tumors. Recently, there have been comprehensive reviews summarizing the involvement of tRF in tumor initiation and progression, elucidating the specific roles played by diverse tRF species across various malignancies [22, 28]. Here, we will specifically discuss the impact of tRNA methylation on cancer progression through the involvement of tRFs.

The relationship between tRNA methylation and tRFs in cancer cells is intricate and plays a crucial role in regulating cancer cell survival. As previously mentioned, the methylation modification of tRNA acts as a protective mechanism against nuclease-mediated cleavage, thereby ensuring tRNA stability and preventing the generation of tRFs. For instance, studies have demonstrated that the depletion of DNMT2, an m^5^C methyltransferase responsible for tRNA modification, reduces tRNA m^5^C levels and increases the production of tRFs [108, 109]. Loss of tRNA m^5^C methylation in NSUN2-deficient cells has also been observed to enhance ANG-mediated tRNA cleavage, resulting in the accumulation of 5’ tiRNA molecules [110]. In addition, the knockdown of TRMT2A resulted in decreased m5U modification levels and an upregulation of ANG activity. This led to cleavage near the tRNA anticodon, facilitating the generation of 5’ tiRNAs, such as 5’ tiRNA-GlyGCC and 5’ tiRNA-GluCTC, among others. This observation highlights the potential role of m^5^U54 as a protective marker against tRNA cleavage [111]. Recent studies have also demonstrated that depletion of METTL1 results in the loss of m^7^G tRNA methylation and facilitates the generation of a novel class of small non-coding RNAs derived from 5’tRNA fragments, which exert tumor cell growth arrest through translation repression mediated by guidance from 5’ tRNA-derived small RNAs [112]. It can be seen that the function of tRFs generated by tRNA cleavage due to the lack of methylation is more inclined to inhibit tumorigenesis. However, tRFs generated from the cleavage of tRNA by some demethylases do not support this hypothesis. For example, the demethylases ALKBH1 and ALKBH3 possess the capability to remove the methyl group from m1A in tRNAs, leading to increased susceptibility to angiogenin cleavage and subsequent generation of tRFs [76, 113]. As previously mentioned, both ALKBH1 and ALKBH3 have been implicated in promoting tumor cell growth. These examples illustrate that the impact of tRFs generated through tRNA methylation regulation on tumor cell survival is intricate. Although some attention has been given to tRNA cleavage, the characterization of different types and functions of tRFs following tRNA cleavage under various conditions remains insufficiently explored. Further investigation is warranted.

## Mechanisms of tRNA methylation modification regulation of tumor cell survival

**Fig. 5:**
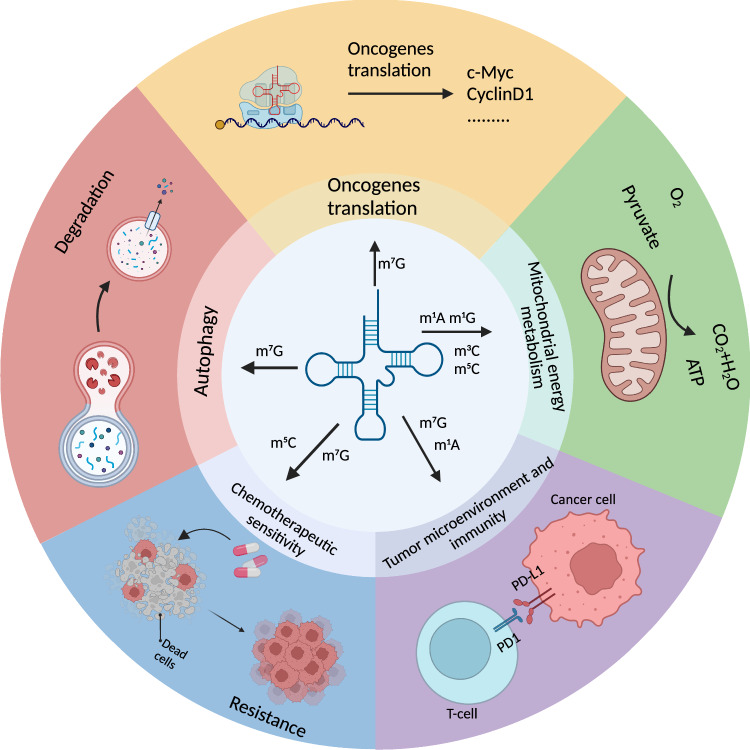
The biological function of tRNA methylation in cancer cells. Oncogene translation: tRNA methylation modification, has been demonstrated to enhance the efficiency of protein translation, particularly for oncogenes, thereby facilitating cancer cell proliferation and growth. Mitochondrial energy metabolism: Methylation modifications of mitochondrial tRNA can promote the synthesis of the mitochondrial respiratory chain, enhancing mitochondrial energy metabolism levels. Autophagy: tRNA methylation can alter the autophagy status of cancer cells to support their survival. Chemotherapeutc sensitivity: The methylation of tRNA can enhance the translation of proteins associated with chemotherapy resistance, thereby facilitating cancer cell chemotherapy resistance. Tumor microenvironment and immunity: tRNA methylation in T cells can induce T cell activation and enhance the sensitivity of cancer cells to PD1/PD-L1. Created with BioRender.com.

The processes of tRNA and protein synthesis are universally observed in all cellular systems. However, the genome shows substantial variation in its preference for specific codons in its coding sequence. The mechanism of tRNA-mediated translational preference, although unclear and still controversial, may reflect the selection of tRNA modifications for translation efficiency and accuracy in tumor cells, while translation speed shows a significant correlation between codon usage preference and tRNA abundance, highlighting mRNA codon usage as an optimizing factor for overall cellular efficiency [114]. Translation preference mediated by tRNA methylation modification has been confirmed to be closely related to tumor cell proliferation and differentiation [115, 116], and the preference of some rare codons for oncogene translation has been shown to influence KRAS-driven tumorigenesis [117]. At the same time, high expression of tRNA and high level of tRNA modification is often associated with high expression of corresponding modifying enzymes [4]. Alterations in translation processes involving tRNA methylation are critical in mediating alterations in tumor migration and invasive phenotypes, including EMT, cellular material recycling, metabolic reprogramming, resistance to chemotherapeutic agents, and the tumor microenvironment [31, 118]. It can be concluded that tRNA methylation modification determines cancer cells’ fate (as shown in Fig. 5).

### Promotion of oncogene translation

Dysregulation of gene expression is considered a significant biomarker for cancer cells. However, it should be noted that the correlation between mRNA and protein levels in cancer is often weak due to substantial variations in mRNA translation efficiency across different genes [119]. Cancer cell processes are subject to a range of endogenous metabolic and environmental stresses, which can cause cell damage, and stress, and disrupt the physiological conditions necessary for cancer cell growth [120]. Cancer cells usually exhibit dysregulation of tRNA expression, coupled with a significant increase in RNA Pol III activity that ensures a sufficient amount of tRNA to maintain the rate of protein synthesis required by cancer cells in response to cellular stress [121]. Moreover, tRNAs play a pivotal role in the process of protein synthesis and undergo extensive methylation, thereby augmenting their stability. Consequently, this process amplifies its impact. For instance, a recent study has demonstrated that numerous genes that promote the cell cycle and tumor metastasis are abundant in AGA codons, such as CDK4, HMGA2, and others, and the overexpression of these AGA codon-rich genes, which happens due to METTL1-dependency, has been discovered in several tumors like AML, glioblastoma, and liposarcoma that may as well overexpress METTL1. METTL1 changes ArgTCT4-1, a tRNA that reads the AGA codon, leading to the promotion of the translation of cellular oncogenes in these tumors [4]. This indicates that the effective transcription of tRNAs, which have high levels of methylation modification, boosts the synergistic translation of oncogenes in tumor cells. In the case where the METTL1, m^7^G tRNA methyltransferase, was knocked down, the oncogene mRNA translation process was blocked. This blockage inhibited the proliferation of tumors by blocking the cell cycle and the EGFR pathway [116]. It is worth noting that certain oncogenes, such as c-Myc and Ras/ERK, have been observed. Some of these oncogenes, including c-Myc and Ras/ERK, exhibit the potential to promote tumor growth by selectively inducing the expression of specific tRNAs through RNA Pol III complexes, thereby facilitating tumor growth and invasion [122, 123].

### Regulation of mitochondrial energy metabolism

Reprogramming of tumor energy metabolism represents a burgeoning hallmark and research focus in the field of cancer, exerting regulatory control over the metabolic modalities of tumor cells to drive their rapid growth and proliferation [124]. Normal cells derive energy by initiating glycolysis in the cytoplasm followed by oxidative phosphorylation (OXPHOS) in the mitochondria under aerobic conditions. However, tumor cells exhibit distinct metabolic pathways compared to normal cells. In the 1920s, Otto Warburg and colleagues made an observation that tumors displayed heightened glucose uptake in comparison to surrounding tissues. Furthermore, even under sufficient oxygen availability, tumor cells preferentially undergo glycolysis leading to lactic acid production - a phenomenon known as aerobic glycolysis. Conversely, normal cells predominantly rely on oxidative phosphorylation for ATP generation through aerobic respiration [125]. The Warburg effect is recognized as a key factor in supporting the uncontrolled proliferation of tumor cells. Tumor cells experience infinite proliferation and therefore require a much faster energy supply. Although glycolysis-produced ATP is less efficient per glucose molecule, it produces ATP at a faster rate than oxidative phosphorylation [126]. Recent studies have found that although the Warburg effect promotes tumor cell growth, tumors also necessitate a high degree of metabolic flexibility to cope with local environmental stresses. The OXPHOS pathway contains over 100 proteins. The mitochondria themselves translate 13 OXPHOS subunits. To translate these essential subunits of the respiratory chain complex, the mitochondrial genome, as a semi-autonomous organelle, contains 22 tRNAs modified by 18 types of RNA at 137 positions [127]. Metastasis of cancer cells induces metabolic heterogeneity, necessitating the reliance on mitochondrial OXPHOS for energy production, while tRNA methylation modifications play a pivotal role in reshaping the mitochondrial metabolism of cancer cells [95]. Suppression of mitochondrial NSUN3-mediated m^5^C tRNA modification in cancer cells effectively impedes the metabolic transition from glycolysis to OXPHOS, thereby compromising their metastatic potential despite retaining some proliferative capacity [95].

In addition, the role of the METTL8 tRNA methyltransferase enzyme in metabolic shifts brought about by mitochondrial tRNA modifications was demonstrated to be significant. Initially, it was postulated that METTL8 played a pivotal role in mRNA modification. However, recent studies have shown that METTL8 did not significantly modify mRNA [64]. Instead, it localizes in mitochondria, interacts with mitochondrial tRNAs, and performs m^3^C32 modification of mitochondrial tRNAs [64]. Deletion of METTL8 compromises the functionality of the mitochondrial respiratory chain and is correlated with both pancreatic cancer cell proliferation and patient survival [64]. METTL8 increases mt-tRNA^Ser (UCN)^ m^3^C32 modification levels in the pancreatic cancer cell line PANC-1, which activates mitochondrial respiratory chain activity [64]. However, in the past, The tRNA modification m^3^C32 on mt-tRNA^Ser(UCN)^ and mt-tRNA^Thr^ has been known for a long time, but the responsible enzyme has remained elusive [128]. Although METTL8 is highly expressed in a variety of cancers, patient survival is only associated with high METTL8 levels in pancreatic cancer. Since an increase in METTL8 is associated with increased respiratory chain activity, the obvious benefit to pancreatic cancer cells may be a better supply of energy, allowing for rapid and aggressive progression [64].

In addition, there are other mt-tRNA methyltransferases, including TRMT5 (an m1G methyltransferase) and TRMT10C (an m^1^A methyltransferase), that have been reported to be associated with liver cancer progression [129, 130]. Meanwhile, TRMT61B, an m^1^A-modifying enzyme of mt-tRNA^leu (UUR)^, which was recently identified as a biomarker and novel therapeutic target for highly aneuploid cancers, has also been found to play an important role in tumor progression, but the effects on mitochondrial pathways have not been thoroughly investigated [131]. However, we are confident that they are also likely to play a critical role in mitochondrial remodeling of the metabolic state of tumors in the environment, and thus it is reasonable to assume that mt-tRNA methyltransferases are a promising therapeutic target for single or combination drugs.

### Autophagy

Macroautophagy/autophagy is an evolutionarily conserved intracellular degradation process, which serves as a crucial mechanism in maintaining cellular homeostasis [132]. Autophagy aids in the elimination of misfolded proteins and damaged organelles, while also supplying vital nutrients for cell survival, particularly during instances of cellular stress [133]. Autophagy is categorized into macroautophagy, microautophagy, and molecular chaperone-mediated autophagy. Among them, macroautophagy (hereafter as autophagy) is the most common and widely studied. Autophagy is a two-sided mechanism in cancer therapy, with an initial autophagy flux inhibiting the formation of cancer cells. However, after cancer cell formation, autophagy can promote their survival in a stressful environment [134]. Gene expression can be dysregulated, resulting in abnormal autophagy in cancer. This process involves multiple steps, including DNA replication, gene transcription, mRNA translation, and post-translational regulation. New reports suggest that protein levels in cancer cells are not directly correlated with mRNA levels and that certain oncogenes are overexpressed in a translational regulatory manner that is closely tied to the development of cancer [135]. In yeast cells, autophagy is facilitated by elevated levels of tRNA modifications and aberrant tRNA expression. The precise mechanism underlying this phenomenon remains elusive; however, it may be linked to nutrient sensing during starvation, mTOR activity modulation, and ATG8 cleavage. Furthermore, the occurrence of protein aggregates has been correlated with specific anticodon defects that subsequently promote autophagy [136]. This activation of autophagy is believed to be advantageous for tumor cell survival and drug resistance [135]. In addition, modifications in tRNA methylation can alter the translation levels of genes related to autophagy, ultimately affecting the autophagy status of tumor cells in diverse conditions. It is important to note that changes in phosphorylation of ULK1 mediated by AMPK and mTORC1 play a crucial role in regulating autophagy dynamics [134]. Interestingly, a recent report indicated that inhibiting METTL1/WDR4-mediated m^7^G tRNA modification significantly decreased mRNA translation efficiency along the mTOR pathway. This led to the inhibition of ULK1 phosphorylation, activating autophagy, and ultimately causing “autophagic cell death” due to excessive autophagy in ESCC cells [137].

These lines of evidence suggest a bidirectional relationship between various tRNA methyltransferases and types of tRNA modifications in autophagy regulation within tumor cells; however, overall, methylation modification of tRNAs tends to promote cancer cell progression. These findings underscore the crucial roles played by tRNA methylation modification and methyltransferases in maintaining autophagic flux in tumor cells.

### The change of tumor microenvironment and immunity

The crucial role of both the innate and adaptive immune systems in host defense against cancer has been firmly established through a multitude of mechanisms, which are propelling the unprecedented advancements in modern cancer immunotherapy [138]. Cancer is a genetically influenced disease characterized by genomic instability, wherein multiple point mutations accumulate and undergo structural alterations during tumor progression [139]. This genetic variation may result in the alteration of tumor cell surface antigens, enabling their recognition by the immune system as foreign antigens and initiating a cellular immune response. Consequently, the pivotal role of the immune system lies in its ability to discern and eradicate malignant cells [140]. However, tumor cells have developed various strategies to evade the immune response, including impaired antigen-presenting mechanisms, heightened negative regulatory pathways, and enlistment of immunosuppressive cell populations. These mechanisms contribute to the failure of the immune system to detect and identify tumor cells hence resulting in tumor cell immune evasion [141]. Therefore, reactivating the immune system to identify and eradicate tumor cells is a prominent area of study. Antigen-specific T cells are crucial in the eradication of tumors. Tumor cells trigger the cancer immune cycle when they release tumor antigens. To achieve optimal activation, co-stimulatory signaling between T cells and APCs is necessary, along with immune checkpoints like PD1 and its ligand, PD-L1, to prevent excessive T cell activation. However, tumor cells can exploit the PD1/PD-L1 property for immune evasion [142, 143]. Tumor immunotherapy has made significant clinical progress in recent years, particularly with the utilization of over-the-counter cell transfer therapy and ICB. Among these advancements, ICB targeting the PD1/PD-L1 axis is a prominent example that has been extensively used to treat multiple types of solid tumors [144]. However, the application of PD1/PD-L1 as a therapeutic target in certain cancers often gives rise to resistance, thereby leading to cancer recurrence [144]. Interestingly, recent studies have unveiled the pivotal role of tRNA methylation modifications in shaping the tumor immune microenvironment through translational regulation. Notably, certain advanced cancers, such as cholangiocarcinoma, exhibit suboptimal response to ICIs due to the presence of immunosuppressive cells like MDSCs. METTL1 enhances CXCR2 translation through m^7^G modification of tRNAs, leading to increased accumulation of PMN-MDSCs and resulting in decreased drug sensitivity of PD1/PD-L1 [145]. This study forms the foundation for a therapeutic plan that involves the augmentation of ICIs with PD1/PD-L1 by METTL1/CXCR2 combination inhibitors. Additionally, enhanced activation of T-cells can attenuate resistance to PD1/PD-L1 blockade. T cells undergo substantial phenotypic changes from a quiescent state to a hyperactive state, necessitating de novo protein synthesis through transcription and translation processes. In this intricate cascade, tRNA methylation modifications play an indispensable role. For example, the tRNA m^1^A modification has been revealed to function as a translational checkpoint and accelerator, promoting T cell expansion [146, 147]. Recently, the up-regulation of tRNA m^1^A58 methyltransferases TRMT61A and TRMT6 has been discovered when T cells exit the quiescent state. The resulting m^1^A modification is specific to early-expressed subpopulations of tRNA, thereby enhancing translational efficiency for the rapid and essential synthesis of MYC and a distinct set of pivotal functional proteins that facilitate T-cell proliferation and function, while concurrently augmenting PD1/PD-L1 sensitivity [118]. It is reasonable to infer that tRNA methylation modifications and methyltransferases play significant roles in the tumor microenvironment, indicating that the latter can be potential therapeutic targets in future cancer treatments based on immunotherapy. The use of these methyltransferases should complement current immunotherapy regimens to create a promising therapeutic modality.

### Chemotherapeutic sensitivity

Chemotherapy drugs are a potent weapon in the fight against tumors. Nonetheless, drug resistance within cancer cells is a prevalent factor in the failure of chemotherapy. Consequently, improving chemosensitivity has gained crucial importance in cancer research. Generally, cancer cells exhibit resistance, which can be categorized as either innate resistance, characterized by an immediate non-response to drugs, or acquired resistance, characterized by a gradual desensitization of cancer cells to therapeutic drugs during the treatment course. Cancer cells develop drug resistance through various mechanisms, such as increased drug efflux and reduced drug uptake, lost drug efficacy, target mutations, and modified signaling pathways within the cancer cell [148]. Continued use of chemotherapeutic drugs accumulates DNA damage and increases the likelihood of genetic mutations. Consequently, a small fraction of tumor cells is screened out, leading to the evolution of resistance against chemotherapeutic regimens [149]. For example, epidermal growth factor receptor (EGFR) is an oncogenic receptor tyrosine kinase that links extracellular signals to the control of cell survival, growth, proliferation, and differentiation, and various drugs targeting EGFR have been developed and marketed [150]. However, the use of EGFR-TIK frequently leads to mutations in EGFR, including T790M, L1196M, and T529N, and activation of the autophagy pathway, resulting in drug resistance in cancer cells [151, 152]. To tackle this challenge, researchers have investigated the utilization of drug combinations, such as dual blockade of oncogenic signaling pathways, multimodal inhibition strategies, intermittent dosing regimens, and alternating dosing schedules [152]. Importantly, when multiple chemotherapeutic agents are used simultaneously, it can result in increased drug toxicity. A recent study has shown that treating ovarian cancer with a sequential combination of PARP and WEE1 inhibitors can effectively minimize adverse drug reactions while maintaining efficacy. The study found that ovarian cancer cells respond differently to this treatment due to variations in replication pressure compared to normal cells [153]. However, it remains to be ascertained whether these enhanced outcomes endure when patients undergo this treatment regimen for an extended duration.

Intensive studies conducted in recent years on tRNA modification and tRNA modifying enzymes have revealed a crucial role in mediating drug resistance in tumors. An earlier study found that knockdown of NSUN2 and METTL1 heightened 5-fluorouracil (5-FU) susceptibility in HeLa cells. The study also identified that NSUN2 and METTL1 were accountable for m^5^C and m^7^G alterations of tRNAs in HeLa cells, respectively. Nevertheless, this study did not detect any influence of NSUN2 on HeLa cell proliferation [154]. Although NSUN2 was confirmed to play an important role in various cancers in later extensive studies, it has been shown that tRNA methylation modifications may have a wide and significant impact on drug resistance in cancer cells [94]. A recent study revealed a significant up-regulation of the m^7^G tRNA methyltransferase METTL1/WDR4 in HCC cells upon lenvatinib treatment, leading to dysregulation of tRNA expression abundance. Additionally, lenvatinib-induced METTL1/WDR4-mediated modification of the m^7^G tRNA facilitates the translation of EGFR and its associated pathway, thereby ultimately eliciting drug resistance in HCC cells [155]. Another study demonstrated that the reduction of METTL1/WDR4 hinders the translational efficacy of the oncogene lysyl oxidase-like 2 (LOXL2), which is a cancer-causing enzyme that promotes the cross-linking of elastin and collagen in the extracellular matrix. Additionally, it enhances the sensitivity of chemotherapeutic drugs, such as doxorubicin in osteosarcoma treatment [156].

Cancer cell resistance occurs for a wide range of complex reasons, including transcriptional and translational activation of oncogenes, enhanced autophagy, and mutations that result in reduced efficacy or even ineffectiveness of chemotherapeutic drugs. Several recent studies have suggested that the regulation of tRNA methylation modification and methyltransferases has the potential to hinder the translation of oncogenes linked to cell proliferation. Furthermore, disrupting the process of tRNA methylation may be more effective in reducing the drug inefficacy caused by specific activating mutations in oncogenes, thus increasing the sensitivity of the combination of chemotherapeutic drugs.

## Therapeutic strategies via targeting tRNA methylation modification process

**Fig. 6:**
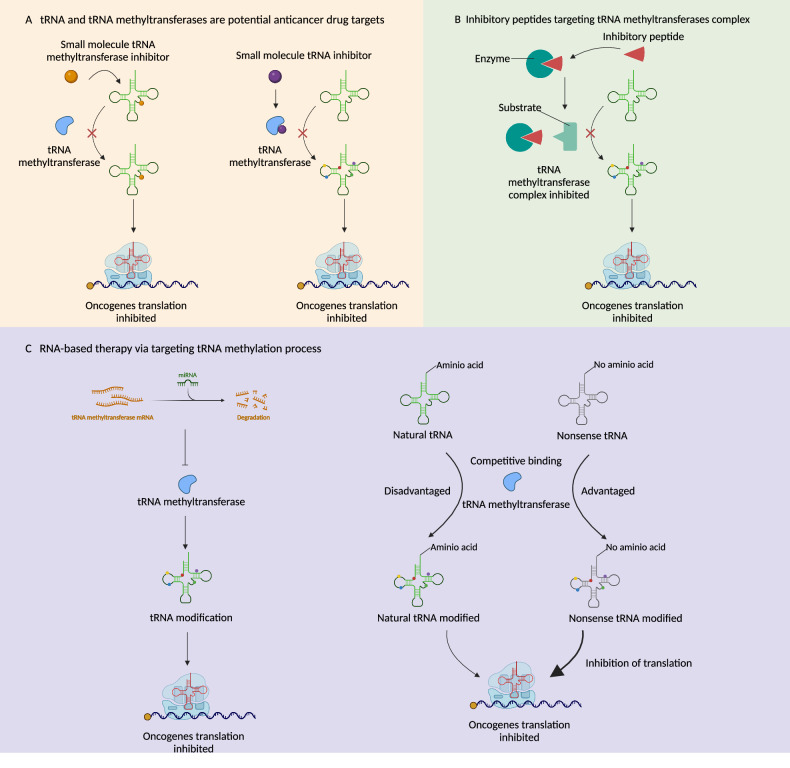
Potential cancer therapeutic strategies based on the tRNA methylation process. **A** The tRNA methylation process can be effectively inhibited by small molecule compounds through direct targeting of tRNA methyltransferases or direct binding to the tRNA methylation site. **B** Inhibitory peptides inhibit the tRNA methylation process by competitively inhibiting the assembly of the tRNA methyltransferase complex. **C** RNA-based therapy via targeting tRNA methylation process involving screening microRNAs to target tRNA methyltransferase or nonsense tRNA as substrates, aiming to decrease the natural level of tRNA modification. Created with BioRender.com.

In this part, we categorize our cancer treatment strategies based on the tRNA methylation modification process into the following sections:

(1) Design and screening of small molecule drugs via targeting tRNA methyltransferases and demethylases. (2) Delivery of therapeutic RNAs such as siRNAs, miRNAs and circRNAs to regulate intracellular tRNA methyltransferase expression. And design of suppressor-tRNAs (stRNAs) for maintenance of tRNA homeostasis and suppression of oncogene mRNA translation. (3) Blocking of the tRNA methyltransferase and its cofactor interactions to inhibit the level of tRNA methylation (As shown in Fig. 6). Potential tRNA methylation-based drugs are summarized in Table 2.

**Table 2:** Prospective pharmaceuticals targeting tRNA methylation in cancer treatment.

Type	Protential representative drug	Taget	Cancer	Protential mechanism	Reference
Small molecular compounds	doxorubicin	tRNA	Broad-spectrum anti-cancer	Directly binds to tRNA	[197]
	KP1019				[198]
	aristololactam-β-D-glucoside and daunomycin				[199]
	Vincristine and vinblastine				[164]
	cisplatin				[200]
	sanguinarine				[201]
	mitoxantrone				[202]
	chelerythrine				[203]
	sinefungin	DNMT2		Inhibition of DNMT2 activity	[159]
	comp 1.4				[159]
	comp 1.4	NSUN6		Inhibition of NSUN6 activity	[159]
	Adeonosin mimics comp 5	METTL1		Inhibition of METTL1 activity	[204]
RNA-based drug	miR-241	TRMT61A	/	Targeting TRMT61A mRNA	[166]
	stRNA-arg	E-cadherin nonsense mutant mRNA	Hereditary diffuse gastric cancer	Recovered full-length E-cadherin expression	[205]
	tRNA^Ser(AAU)^	translation process	Breast cancer	Inhibition of the translation process	[206]
Inhibitory peptides	/	tRNA methyltransferase complex	/	Inhibits the binding of the tRNA methyltransferase complex	Temporarily not available literature

### tRNA and tRNA methyltransferases are potential anticancer drug targets

Small-molecule drugs have traditionally been the go-to method for treating diseases. The development of small molecule drugs commences with identifying the target of function and screening lead compounds. Unlike specific cancer drivers, structures of RNA-modifying enzymes are not typically altered in cancer [96]. Furthermore, multiple studies have demonstrated the link between tRNA modification, protein synthesis rates, cancer progression, and cell differentiation. Additionally, cancer cells have shown a heightened reliance on these enzymes [157]. Therefore, targeting tRNA methyltransferases is a rational strategy for developing new anticancer drugs [158]. More importantly, as previously mentioned tumor cells exhibit higher levels of tRNA methyltransferase-mediated methylation modification of tRNAs compared to normal cells. Therefore, targeting tRNA methyltransferase with small-molecule compounds designed to inhibit its activity may offer a potential therapeutic strategy for treating tumors. There is promise in achieving a significant inhibitory effect on the growth and metastasis of cancer cells by affecting tRNA modification, while limiting harm to normal cells to reduce drug toxicity during systemic chemotherapy.

Previously, NSUN3 was found to be involved in the modification of tumor mitochondrial m^5^C to regulate tumor cell metastasis, but as an independent enzyme, it exclusively promotes mitochondrial m^5^C formation [95]. This indicates NSUN3 as a possible antitumor drug target. Designing small molecule drugs against this target could potentially impede tumor cell metastasis and improve patient prognosis. In addition, chemical space virtual screening of tRNA methyltransferases DNMT2 and NSUN6 has been studied [159]. Given the potential significance of the tRNA methylation process in disease, drug design centered on tRNA methyltransferases remains a subject of interest among researchers, despite the challenging protein structure associated with drug development.

Although excellent reviews have highlighted the potential of RNA-modifying enzymes as effective drug targets [160], a notable instance is the methyltransferase METTL3, which facilitates the m6A methylation of mRNA. Its inhibitor, STM2457, is presently being evaluated for the treatment of acute myeloid leukemia [161]. However, exploring RNA-modifying enzymes as potential drug targets is still in its infancy, and many issues need to be addressed, including small molecule selectivity, drug toxicity, bioavailability, and stability.

Several studies have demonstrated the direct binding of specific chemotherapy medications or their drug metabolites to tRNA molecules, highlighting their potential as chemotherapeutic agents capable of interacting with tRNAs. For instance, Doxorubicin-tRNA binding takes place through both major and minor grooves; however, the tRNA’s conformation remains the same. Such instances offer evidence that chemotherapeutic agents and metabolites can interact with tRNAs directly [162]. Tamoxifen and its metabolites can bind to multiple sites on tRNA without changing its conformation, but they can induce tRNA aggregation [163]. Similarly, Vincristine also can directly interact with tRNA [164]. The impact of drug-tRNA binding on tRNA aggregation is not yet fully understood. However, highly probable that this process reduces the amount of tRNA that enters the ribosome, thereby disrupting protein synthesis in cancer cells. These studies propose that tRNA molecules can also serve as direct targets for drug screening, with drug-specific tRNA binding able to hinder tRNA methylation sites, induce tRNA degradation or aggregation, and weaken the translational ability of oncogenes in cancer cells.

### RNA-base therapy via targeting tRNA methylation process

Non-coding RNA (ncRNA) constitutes a crucial genomic component, encompassing non-coding transcripts that lack protein-coding capacity but possess inherent biological functionality at the RNA level. Diverse therapeutic approaches exploiting ncRNAs have been developed, including antisense oligonucleotides (ASOs), small interfering RNAs (siRNAs) and short hairpin RNAs (shRNAs), microRNAs, therapeutic circulating RNAs (circRNAs), and CRISPR-Cas9-based gene editing. ncRNAs, specifically tRNAs, have demonstrated significant involvement in the proliferation and metastasis of cancer cells, alongside tRNA methylation. As previously mentioned, dysregulation of tRNAs and aberrant modifications are prevalent across various cancer types, highlighting the immense potential for employing tRNA-based therapeutic strategies against cancer.

Gene therapy holds immense potential as a curative intervention for a multitude of presently incurable diseases. Here, we present two therapeutic strategies employing RNA therapeutics to specifically target the tRNA methylation process in cancer cells. The first RNA therapeutic strategy is based on the targeting of mRNA degradation for the tRNA methyltransferase. Currently, RNAi therapy has a well-developed theory and immense potential. Several siRNA drugs using RNAi technology are either on the market or in clinical trials. RNA technology offers opportunities for selectively targeting and silencing mRNA gene products without requiring an in-depth understanding of target protein structures. Additionally, hard-to-drug proteins that play crucial biological roles can be utilized as intervention and treatment targets [165]. Additionally, endogenous miRNA screening also enables precise targeting of tRNA methyltransferases. For example, miRNA-214 inhibits TRMT6/TRMT61A mRNA, a tRNA m^1^A methyltransferase, to reduce cell proliferation. Moreover, TRMT61A exerts inhibitory effects on cell proliferation by modulating the PI3K/AKT/mTOR and ErbB signaling pathways, while simultaneously promoting cancer cell proliferation in gastrointestinal tumor cells [166, 167].

Another strategy is to control the translation process by using synthetic tRNA molecules. The Nonsense-Mediated mRNA Decay (NMD) pathway degrades certain mRNAs with premature termination codons, but not all. The most important cancer-related genes are tumor suppressor genes (TSGs), and NMD can block TSG function, including that of TP53, RB1, or PTEN, resulting in uncontrolled cell growth [168]. Therefore, inhibition of NMD may be an effective strategy for cancer treatment. It has been demonstrated that deploying synthetic suppressor-tRNA (stRNA) can effectively circumvent the premature termination of mRNA translation provoked by nonsense mutations. The stRNA shares significant sequence homology with natural tRNA, but its anticodon can specifically identify termination codons (UAG, UGA, UAA) via base pairing. When the ribosome encounters an early termination codon during translation, stRNA inserts the corresponding amino acid into the peptide chain, and producing a biologically functional full-length protein [169]. This study proves the possibility of the utilization of artificially modified tRNAs. This demonstrates the possibility of utilizing artificially modified tRNAs as a potential disease treatment drug. Can we design an artificial nonsense tRNA based on this principle to replace the target of tRNA methyltransferase, thereby attenuating the level of modification in normal target tRNA or inducing premature translation termination of oncogenes, ultimately impeding cancer cell growth? Currently, several pharmaceutical companies are developing tRNA-based cancer gene therapy [170]. However, tRNA-based therapies, as an emerging RNA technology, are still in the nascent stages of development, and it remains uncertain whether they confer superior advantages over existing small molecule drugs, immunotherapy or gene editing therapies. Moreover, with increasing academic and industry endeavors dedicated to exploring tRNA therapy, its validation in terms of safety and efficacy would undoubtedly unlock a plethora of opportunities for cancer gene therapy.

### Design of inhibitory peptides targeting tRNA methyltransferases

Protein-protein interactions (PPIs) mediate many important cellular functions and regulatory pathways. For example, the TRMT6-TRMT61A methyltransferase complex can mediate tRNA m^1^A modification in hepatocellular carcinoma cells [56]. The METTL1/WDR4 complex is capable of facilitating tRNA m^7^G modifications in several types of cancer cells [171]. The TRMT112-THUMPD3 complex is able to facilitate tRNA m^2^G modifications in cells [172], and so on. The interaction between these tRNA methyltransferase complexes plays a pivotal role in the process of tRNA methylation. Therefore, effectively inhibiting this process can be achieved by disrupting the interactions among these tRNA methyltransferase complexes. Peptides offer a highly specific and potent means to target tRNA methyltransferase complexes binding, reducing off-target effects and ensuring enhanced safety. The aforementioned solution offers a viable resolution to the underlying issue at hand [173]. Although there have been limited investigations on peptide drugs targeting methyltransferase complex interactions, certain studies have endeavored to develop peptide-based therapeutics by elucidating the molecular mechanisms in cancer cells. For example, a study showed that peptides targeting and blocking TRIB3-EGFR interaction can improve NSCLC sensitivity to chemotherapeutic drugs. Additionally, EGFR strongly correlates with NSCLC progression and drug resistance, while TRIB3 boosts EGFR stability, so inhibits the TRIB3-EGFR interaction to degrade EGFR, eventually decelerating NSCLC progression [174]. Another study showed that disrupting the interaction of FAM83A with β-catenin using inhibitory peptide to prevent pancreatic cancer progression, and FAM83A directly binds to β-catenin to inhibit β-catenin phosphorylation thereby promoting β-catenin entry into the nucleus, enhancing the transcription of Wnt downstream target genes and promoting pancreatic cancer progression [175]. The success of these cases validates the feasibility of designing peptide therapeutics that selectively target tRNA methyltransferase complexes. However, despite the large number of published studies on peptide drugs and the computational ability to predict inhibitory peptide structure, there is still no universal guideline for peptide drug design [176]. In summary, the key constituents of a polypeptide therapeutic encompass a cell-penetrating peptide that facilitates endocytosis for intracellular cargo delivery; Stapled peptides designed to disrupt protein-protein interactions; and a linker peptide, serving as a flexible sequence enabling the connection between the cell-penetrating peptide and stapled peptide while preserving the spatial conformation of the peptide against steric hindrance [176–178]. Peptide drugs exhibit excellent target specificity and rapid development, yet their application in pharmacology is constrained by limited oral bioavailability, compromised metabolic stability, inadequate membrane permeability, and swift clearance. Consequently, further chemical modifications are imperative to enhance their efficacy. Unquestionably, peptide-based drugs targeting the tRNA methyltransferase complex hold promising potential for cancer treatment.

## Prospects

tRNA is one of the most richly variety of post-transcriptional modifications of RNA type. The role of tRNA modifications in translational regulation has been progressively elucidated with the development of high-throughput sequencing and mass spectrometry. Among all types of tRNA modifications, tRNA methylation holds paramount importance and has been extensively investigated in the field of RNA research. The pivotal role played by tRNA methyltransferases in regulating gene transcription and translation through the process of tRNA methylation is instrumental in governing diverse intricate mechanisms underlying malignant tumor progression, encompassing proliferation, metastasis, chemotherapy resistance, metabolic reprogramming, and immunity. Furthermore, the upregulation of diverse tRNA methyltransferases and elevated levels of tRNA methylation in cancer cells are frequently observed to meet the heightened demands for protein synthesis, thus indicating the potential development of tRNA methylation as a promising tumor marker. Some tRNA methyltransferases have highly specific modifications targeting only specific tRNA sites, and these tRNA methyltransferases themselves are hard to mutate. Therefore, designing drugs that specifically target the tRNA methylation process in tumor cells can precisely regulate the production of specific tRNAs or tRFs, reducing the impact on other RNA molecules. tRNA methylation, as an emerging therapeutic target, can offer new treatment options for refractory cancers. By targeting the tRNA methylation process in tumor cells, multiple downstream biological processes can be affected, including protein synthesis, gene expression, and stress response. This approach provides various therapeutic benefits for patients. Moreover, compared to traditional broad-spectrum chemotherapy drugs, therapies targeting tRNA methylation may reduce toxicity to normal cells and lower adverse reactions. Overall, the tRNA methylation process is a very promising therapeutic target. Therefore, we suggest several effective therapeutic options that are based on the tRNA methylation process. These options encompass small-molecule drug screening, RNA therapy, inhibitory peptide therapy, as well as exploring synergistic combinations with traditional chemotherapy agents. Although tRNAs were discovered as early as 1965, the tRNA molecule continues to exhibit numerous novel functions up to the present day. However, the role of tRNA methylation in cancer cells remains unclear, as it involves complex intracellular regulatory networks and is difficult to precisely control. Meanwhile, since tRNA and tRNA methyltransferases play roles in various cellular processes, targeting tRNA methyltransferases may lead to unforeseen off-target effects and perturb normal cell function. Additionally, similar to conventional therapies, cancer cells might develop resistance against tRNA methylation-targeted treatments by modulating tRNA modifications or activating compensatory mechanisms. Moreover, our understanding of the long-term effects and potential side effects of targeting tRNA methylation is limited, highlighting the need for further clinical research and validation. The role of tRNA in tumor cells remains largely elusive, and depending on the cancer type and preference for modification by tRNA methyltransferase, tRNA methylation may also exhibit tumor-suppressive effects. Therefore, when designing drugs targeting the process of tRNA methylation, it is crucial to consider the specific function of tRNA methyltransferases in different tumors for precise intervention.
